# Germline BRCA2 Pathogenic Variant in Metaplastic Breast Carcinoma with Heterologous Mesenchymal Differentiation: A Case Report and Literature Review

**DOI:** 10.3390/reports9030255

**Published:** 2026-08-05

**Authors:** Alice Arduini, Rita Polati, Giulio Luigi Bonisoli, Sokol Sina

**Affiliations:** 1 Section of Pathology, Department of Diagnostics and Public Health, University of Verona, 37134 Verona, Italy; 2 Section of Internal Medicine B, Department of Medicine, University of Verona, Piazzale L.A. Scuro 10, 37134 Verona, Italy

**Keywords:** breast cancer, metaplastic carcinoma with heterologous mesenchymal differentiation (MCHMD), BRCA2 mutation, case report

## Abstract

**Background and Clinical Significance**: Metaplastic breast carcinoma (MBC) is a rare type of breast tumor with various subtypes. MBCs are typically high-grade and exhibit a particularly aggressive behavior, with a significant propensity for recurrence and specific chemoresistance, especially in neoadjuvant settings. One of its high-grade variants is the metaplastic carcinoma with heterologous mesenchymal differentiation (MCHMD). At present, the literature regarding the genetic predisposition of MBC and its connection with BRCA2 is limited. Hence, we present a rare case of a 51-year-old patient with a germline BRCA2 pathogenic variant affected by MCHMD. **Case presentation**: A 51-year-old Caucasian woman with a family history of breast cancer noticed a lump in her right breast. A needle biopsy of the mass resulted in a diagnosis of poorly differentiated (G3) invasive ductal carcinoma, associated with a dominant component of pleomorphic carcinoma with osteoclast-like cells. After surgery, the pathological report diagnosed a metaplastic carcinoma of the breast with heterologous mesenchymal differentiation (MCHMD) according to the WHO 2019 classification. Genetic testing revealed the presence of the pathogenic variant c.9676del of the BRCA2 gene. **Conclusions**: We report, to the best of our knowledge, the first case of a BRCA2 mutation in a woman with metaplastic carcinoma of the breast with heterologous mesenchymal differentiation.

## 1. Introduction and Clinical Significance

Metaplastic breast carcinoma (MBC) is a heterogeneous group of rare subtypes accounting for 0.2–5% of all breast cancer. It typically affects women over 50 years of age, with a mean age at presentation of 55–60 years, and is associated with a worse overall prognosis than other breast cancer subtypes [1,2].

Histologically, MBCs are characterized by an admixture of neoplastic glandular epithelium and areas displaying non-glandular components with mesenchymal and/or sarcomatoid differentiation. According to the latest edition of the World Health Organization (WHO) Classification of Tumors (2019), MBC encompasses six distinct histotypes [3]:

- Low-grade adenosquamous carcinoma;
- Fibromatosis-like metaplastic carcinoma;
- Spindle cell carcinoma;
- Squamous cell carcinoma;
- Metaplastic carcinoma with heterologous mesenchymal differentiation and mixed metaplastic carcinomas.

Apart from the low-grade subtypes (low-grade adenosquamous carcinoma and fibromatosis-like metaplastic carcinoma), MBCs are typically high-grade tumors characterized by absence of hormone receptors and HER2 expression, along with elevated rates of p53, CK5/6, Ki-67, and EGFR expression [4]. This molecular profile, combined with insensitivity to chemoradiotherapy and late diagnosis, contributes to the poor prognosis associated with these tumors, in which surgery remains the gold standard treatment [4,5,6,7]. As Corso et al. reported, neoadjuvant chemotherapy fails in about 40% of MBC patients with consequent disease progression, while 60% of MBC patients fail to achieve a response following chemoradiotherapy [7].

Given the rarity of MBC, data on genetic predisposition to this breast cancer subtype remain limited. Only a few studies in the literature have investigated germline mutations of BRCA1/2 mutations in MBC patients. Germline BRCA1/2 mutation testing is the recommended approach for identifying patients who may benefit from poly (adenosine diphosphate [ADP]-ribose) polymerase (PARP) inhibitors. Accordingly, current guidelines recommend the use of PARP inhibitors for treatment of advanced BRCA1/2-breast cancer and, more recently, for patients with high-risk, early-stage, HER2-negative disease [8].

A recent study by Corso G. et al. evaluated 23 patients with MBC, revealing that approximately 65% (15/23) of them carried a germline pathogenic variant (PV); of these, 86.7% (13/23) involved BRCA1. Notably, no MBC cases were observed among individuals with BRCA2 mutations [9].

Among the MBC histotypes, metaplastic breast carcinoma with heterologous mesenchymal differentiation (MCHMD) represents one of the rarest and least characterized variants, accounting for 0.08–0.2% of all breast cancers [2,10]. MCHMD is defined as a high-grade tumor characterized by distinct metaplastic components (e.g., chondroid, osseous, rhabdomyoid, and neuroglial) admixed with carcinomatous areas showing glandular differentiation, tubular structures, solid clusters, and/or foci of squamous differentiation [2,10]. The limited evidence currently available regarding the origin, pathogenesis, and biological behavior of this histotype precludes the establishment of a standardized treatment approach and reliable prognostic assessment.

Here we report, to the best of our knowledge, the case of MCHMD associated with a germline BRCA2 pathogenic variant, identified in a 51-year-old patient.

## 2. Case Presentation

In December 2022, a 51-year-old Caucasian woman with a family history of breast carcinoma involving her maternal aunt and paternal cousin, but no family history of ovarian cancer, presented with a self-detected lump in her right breast and was referred to our institution for comprehensive breast evaluation. Her past medical history was notable for a childhood thoracic hemangioma treated with cobalt radiotherapy and toxic multinodular goiter. She had also recently undergone removal of a levonorgestrel-releasing intrauterine device.

Ultrasound examination detected a 2.5 cm nodule with malignant features (U5) in the right axillary extension. Diagnostic cytological examination, performed at an outside institution, was classified as category C5. Core needle biopsy confirmed the presence of an invasive ductal carcinoma, poorly differentiated (Grade 3), with a predominant component of pleomorphic carcinoma containing osteoclast-like giant cells, as well as an additional component of ductal carcinoma in situ (DCIS) with a solid architectural pattern and central comedonecrosis was also observed. Immunohistochemical profile of the invasive ductal carcinoma revealed a triple-negative phenotype (ER-, PR-, AR -, Her-2 score 0) with PDL-1 TPS 0% and a high cell proliferation index (Ki67 30%).

Breast MRI revealed a nodular area of heterogeneous enhancement, measuring approximately 25 mm along the major axis in the right axillary extension, consistent with the expansive lesion previously identified through the biopsy. Multiple enlarged lymph nodes were also identified in the axillary region, the largest measuring 11 mm in major-axis diameter. Cytological examination of the lymph nodes was negative for malignancy. Germline genetic testing identified the pathogenic variant c.9676del in the BRCA2 gene.

In March 2023, the patient underwent a right nipple-sparing mastectomy with sentinel lymph node biopsy, contralateral left prophylactic nipple-sparing mastectomy, bilateral reconstruction, and prophylactic salpingo-oophorectomy.

**Figure 1:**
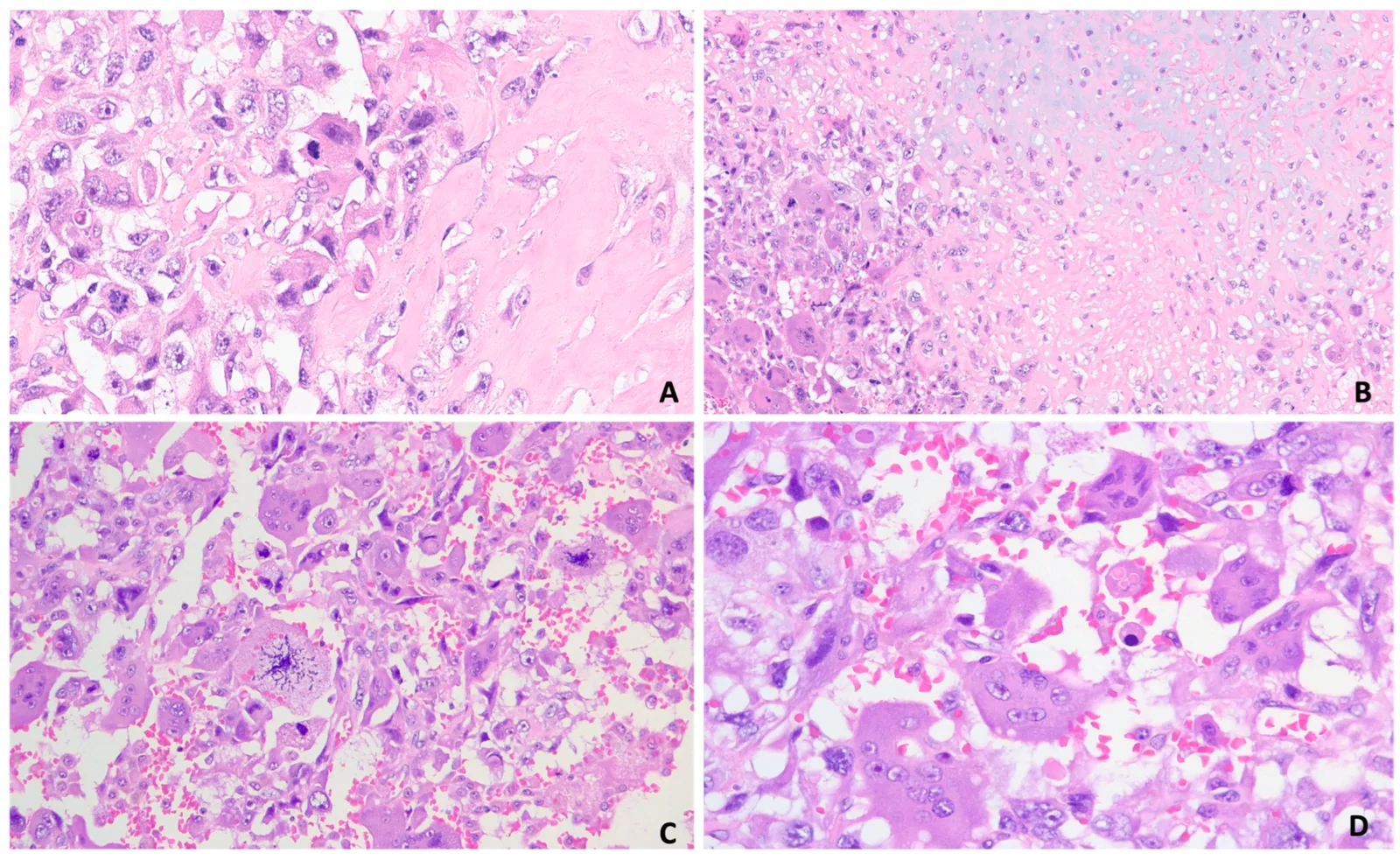
Hematoxylin–Eosin staining. (**A**): Immature bone matrix and osteoclast-like mesenchymal neoplastic cells (Objective ×20). (**B**): Immature bone matrix, chondroid and osteoclast-like mesenchymal neoplastic cells (Objective ×10). (**C**,**D**): Osteoclast-like mesenchymal neoplastic cells and atypical mitoses (panel (**C**) Objective ×20—panel (**D**) Objective ×40).

Gross pathological examination of the right breast specimen showed a brownish mass with a grayish peripheral halo in the outer upper quadrant. The lesion had a firm, woody consistency, measuring 1.2 × 1.0 × 0.9 cm. Microscopic examination demonstrated a tumor composed of an admixture of mesenchymal and epithelioid components consisting of osteoid matrix (95%) with multinucleated giant cells resembling osteoclasts, along with chondroid matrix component (2%), and invasive carcinoma of not otherwise specified (NOS), Grade 3 (3%). An extensive component of ductal carcinoma in situ (DCIS) with a solid architectural pattern and central comedonecrosis was also present. (Figure 1).

**Figure 2:**
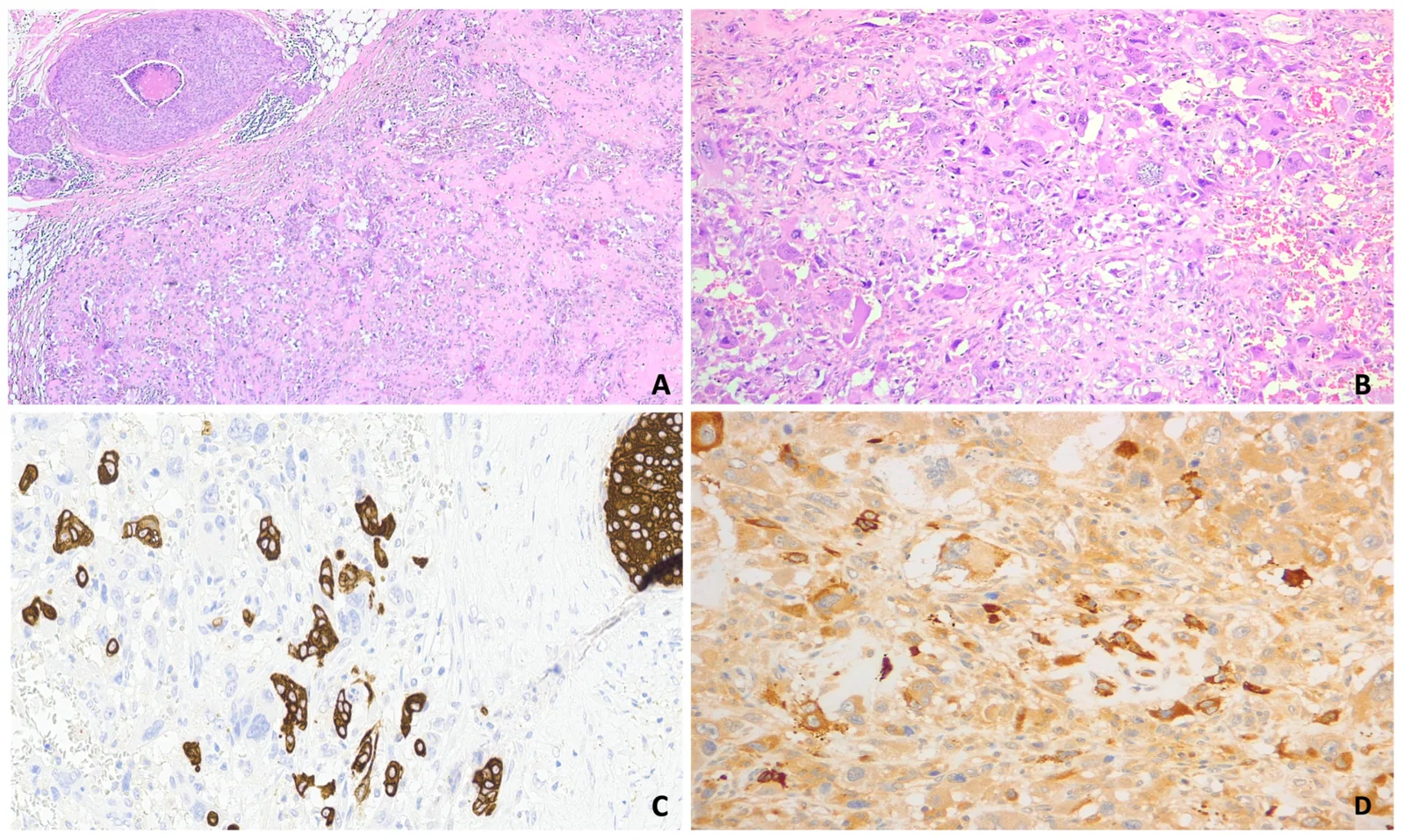
(**A**) Hematoxylin–Eosin (H&E) staining. Immature bone matrix and osteoclast-like mesenchymal neoplastic cells, ductal carcinoma in situ with comedonecrosis (Objective ×10), (**B**): Hematoxylin–Eosin (H&E) staining. Immature bone matrix, osteoclast-like mesenchymal neoplastic cells (Objective ×20), (**C**): immunohistochemistry AE1AE3 positive in IBC NST neoplastic aggregate and negative in osteoclast-like mesenchymal neoplastic cells (Objective ×40) (**D**): immunohistochemistry CD68 positive in osteoclast-like mesenchymal neoplastic cells (Objective ×40).

Immunohistochemical analysis was negative for estrogen and progesterone receptors, androgen receptor, and HER2 (score 0 according to ASCO-CAP guidelines), confirming the triple-negative phenotype of the disease, the cellular proliferation index was 30–40%. (Figure 2).

Based on the histopathological and immunohistochemical findings, a final diagnosis of metaplastic carcinoma of the breast with heterologous mesenchymal differentiation (MCHMD) was established, in accordance with WHO 2019 Classification of Tumors. Sentinel lymph nodes were negative for metastatic cells. According to the AJCC TNM classification, the tumor was staged as pT1cN0M0.

Due to the aggressive biological profile of this tumor histotype, the patient was referred for adjuvant chemotherapy. She received four cycles of EC90 (epirubicin and cyclophosphamide), followed by twelve weekly cycles of carboplatin AUC2 plus paclitaxel, with the final cycle administered in November 2023. Subsequently, one year of adjuvant olaparib was recommended. However, after the first dose, the patient developed grade 3 neutropenia associated with grade 1 mucositis and diarrhea, and she withdrew treatment. Given the germline BRCA2 pathogenic variant identified preoperatively, gynecological surveillance was also recommended as part of the patient’s long-term follow-up protocol. At the time of writing, the patient was alive and had no evidence of disease with a follow-up duration of 43 months.

## 3. Discussion

MBCs are rare neoplasms with biological behavior variables from low to high grades that tend to have large tumor size and more advanced stage. These cancers have a poor response to neoadjuvant systemic therapy, with a pathological complete response (pCR) rate of ~10–17%, making surgical resection the cornerstone of treatment for these tumors. In contrast, our patient presented with an early-stage disease, and she remained disease-free at the time of last follow-up. This suggests that early surgical management combined with systemic therapy can still achieve favorable short-term outcomes in selected cases. Histologically, MBCs comprise different cancer subtypes, including MCHMD, a high-grade rare variant characterized by epithelial and mesenchymal components [10].

In our patient, the tumor showed extensive osteoid matrix with osteoclast-like giant cells (OGC), minor chondroid areas, and a residual invasive carcinoma component, which is consistent with diagnostic criteria for MCHMD according to the WHO classification. OGCs are a rare stromal feature reported only in 0.5–1.2% of breast carcinomas; they are typically associated with luminal A invasive carcinomas, are observed in younger women, and are generally linked to a more favorable outcome. However, current evidence suggests that prognosis is primarily determined by the underlying carcinoma type rather than by the presence of OGCs per se [11].

MBCs, including MCHMD, exhibit a triple-negative phenotype, despite relative gene amplification, and remain poorly characterized at the genomic level [12]. Therefore, there has been growing interest in identifying germline alterations that may contribute to their pathogenesis and help to identify patient that may benefit from targeted therapy. However, data on germline predisposition are particularly scarce.

At the moment, only eight case reports have provided detailed information on germline mutations in MBC patients. In all cases, PVs were identified exclusively in the BRCA1 gene. These reports included: a 25-year-old German woman with metaplastic squamous cell carcinoma carrying the BRCA1 c.181 T>G mutation; a Russian 35-year-old with carcinosarcoma harboring BRCA1 c.5266dup; a 49-year-old Belgian woman presenting with adenosquamous carcinoma and BRCA1 c.66dup; a 22-year-old Pakistani woman with MBC harboring the deleterious BRCA1 c.68_69del variant; a 20-year-old patient with MBC carrying germline mutations in both BRCA1 (c.81-134+del) and TP53; and a 35-year-old woman with carcinosarcoma of the right breast harboring BRCA1 c.4754-4755del; a 39-year-old Japanese woman with invasive breast cancer with chondroid metaplasia and BRCA1 c.188T>A; and a 15-year-old girl whose right-sided breast mass, initially diagnosed clinically as a fibroadenoma, was subsequently found to be stage IIb (pT3N0M0) MBC harboring BRCA1 c.4845G>C and concurrent SMARCA4 variants. This latter case represents the youngest reported case of breast cancer in Pakistan [13,14,15,16,17,18,19,20].

One additional study investigated whether MBCs and uterine carcinosarcomas (UCSs) share similar patterns of genetic alterations, and whether the different histologic components of MBCs and UCSs are clonally related. Using tumor tissue sequencing, authors identified germline variants associated with loss of heterozygosity or a second somatic hit in eight MBCs: BRCA1 mutations in six cases, a BRCA2 variant in one case, and a homozygous BRCA2 deletion in one case [21].

The largest series reported to date was published by Corso et al. from the European Institute of Oncology, who were the first to demonstrate an association between MBC and germline BRCA1 PVs in a well-characterized cohort. Among 5226 patients who underwent germline testing for BRCA1/2, 23 (0.4%) were diagnosed with MBC. Of these, 21 patients (91.3%) underwent comprehensive BRCA1/2 analysis, whereas two (8.7%) were tested for a known familial BRCA1 PV. Of these, 21 patients (91.3%) underwent comprehensive BRCA1/2 analysis, whereas two (8.7%) were tested for a known familial BRCA1 PV. Overall, 15 MBC patients (65.2%) were found to carry a germline PV: 13 in BRCA1 (86.7%), one in TP53 (6.7%), and one in MLH1 (6.7%). For comparison, within the entire cohort of 5226 patients, 610 (11.7%) carried a BRCA1 PV, 498 (9.5%) a BRCA2 PV, and 6 (0.1%) harbored a PV in both genes. Notably, MBC was diagnosed in 2.1% of BRCA1 carriers (13/610), whereas no cases of MBC were identified among BRCA2 carriers (0/498; 0%) [9]. Table 1 summarizes the current literature on the theme.

Against this background, we report what is, to the best of our knowledge, the first documented case of MCHMD in a young woman (51 years old) harboring a germline BRCA2 pathogenic variant (c.9676del).

The BRCA2 c.9676del mutation is located in exon 26 and results in a frameshift caused by the deletion of a single nucleotide at position 9676, thereby altering the gene’s reading frame. The translational frameshift converts a tyrosine codon into an isoleucine codon at position 3226 and creates a premature stop codon at position 23 of the new reading frame (p.Y3226Ifs*23). The resulting nonfunctional truncated protein lacks the final 193 amino acids of the wild-type BRCA2 protein, which are replaced by 22 aberrant amino acids. Consequently, the truncated protein loses several critical functional domains, including nuclear localization signals (NLS1 and NLS2), Cyclin A and RAD51 binding sites, as well as multiple phosphorylation sites [22,23,24,25].

Thus, the physiological function of the BRCA2 protein is lost. The resulting impairment of BRCA2-mediated homologous recombination repair, which is critical for preventing cancer development, promotes genomic instability and is consistent with the established role of BRCA2 in hereditary breast and ovarian cancer predisposition. Accordingly, the c.9676del pathogenic variant has been independently reported in individuals with breast and ovarian cancer by Santonocito et al., Rebbeck et al., and Pilato et al. [26,27,28].

## 4. Conclusions

MBCs are a genetically complex and heterogeneous group of tumors whose molecular landscape remains incompletely characterized, because of their rarity and the limited number of patients included in molecular studies.

The available genomic studies suggest that, although MBCs and TNBCs share frequent TP53 mutations, MBCs exhibit a higher prevalence of mutations affecting PI3K and WNT signaling pathway genes compared to TNBCs.

Moreover, MCHMD appears to lack TERT promoter mutations, suggesting distinct oncogenic mechanisms that may have implications for the development of targeted therapy [29].

From a germline perspective, Corso et al. were the first to establish a significant association between MBCs and germline BRCA1 PVs, reporting an increased prevalence of MBC among BRCA1 carriers, whereas no cases were identified among BRCA2 carriers in their cohort [9]. Against this background, our histologically confirmed case of MCHMD in a patient harboring the germline BRCA2 PV c.9676del expands the spectrum of germline predisposition in this rare histological subtype. Although a causal relationship cannot be inferred from a single case, our findings suggest that BRCA2 mutations may confer a specific, albeit low, risk for MCHMD and warrant investigation in larger prospective series.

Overall, the available literature underscores the need for molecular and germline characterization of MBCs in larger, histologically stratified cohorts. Such studies will be essential to elucidate the pathogenetic mechanisms driving MBC, refining genotype–phenotype correlations, and to identify actionable targets that may support the development of more effective, subtype-specific therapeutic strategies.

## Figures and Tables

**Table 1:** Cases of metaplastic carcinoma of the breast with germline mutations in BRCA1 and the current one with germline mutation in BRCA2.

Patient	Age at Diagnosis	Family History	Gene	Germline Defect	Side	Histology of MBC	Sub-Type	Other Tumors (Age)
Breuer 2007 [13]	25	BC	BRCA1	c.181 T>G p.(Cys61Gly)	Right	Squamous cell carcinoma	TN	Left MBC (28)
Noël 2010 [15]	49	No family history	BRCA1	c.66dup p.(Glu23Argfs*18)	Right	Low-grade adenosquamous carcinoma	TN	Right IDC (36)
Susptisin 2011 [14]	35	OC	BRCA1	c.5266dup p.(Gln1756Profs*74)	Left	Mixed metaplastic carcinoma	TN	
Rashid 2011 [16]	22	No family history	BRCA1	c.68_69del p.(Glu23Valfs*17)	Left	Mixed metaplastic carcinoma	TN	
Bell 2014 [17]	20	BC	BRCA1 TP53	c.81-?_134+?del p.(Cys27*) c.375+ 2 T>C p.(?)	Right	Metaplastic Carcinoma, NOS	TN	Right IDC (20)
Ghilli 2017 [18]	35	BC	BRCA1	c.4754_4755del p.(Pro1585Argfs*36)	Right	Mixed metaplastic carcinoma	TN	Right IDC (35)
Yamashita 2021 [19]	39	BC/OC	BRCA1	c.188 T>A p.(Leu63*)	Left	Mixed Metaplastic Carcinoma	TN	Left IDC (39)
Vohra 2022 [20]	15	No family history	BRCA1 SMARCA4	c.1961dup p.(Tyr655Valfs*18) c.4845 G>C p.(Glu1615Asp)	Right	MCHMD	TN	
Corso 2023 [9]	44	No family history	BRCA1	c.(?_-119)_(80+1_81-1)del p.(?)	Left	Squamous cell carcinoma	TN	Right IDC (40) Left IDC (42)
Corso 2023 [9]	36	No family history	BRCA1	c.798_799del p.(Ser267Lysfs*19)	Left	MCHMD	TN	Right IDC (24)
Corso 2023 [9]	37	BC/OC	BRCA1	c.816_825dup p.(Thr276Alafs*14)	Left	Squamous cell Carcinoma	TN	
Corso 2023 [9]	48	BC	BRCA1	c.2934 T>G p.(Tyr978*)	Right	Squamous cell Carcinoma	TN	Left IDC (30) Right IDC (37)
Corso 2023 [9]	47	BC	BRCA1	c.3904 G>T p.(Glu1302*)	Left	MCHMD	TN	
Corso 2023 [9]	35	No family history	BRCA1	c.3228_3229del p.(Gly1077Alafs*8)	Left	MCHMD	TN	Right IDC (39)
Corso 2023 [9]	52	No family history	BRCA1	c.1088del p.(Asn363Ilefs*11)	Left	MCHMD	TN	
Corso 2023 [9]	47	No family history	BRCA1	c.4186-3156_4357+94dup p.(?)	Right	Spindle cell Carcinoma	TN	
Corso 2023 [9]	40	BC	BRCA1	c.4964_4982del p.(Ser1655Tyrfs*16)	Left	Mixed metaplastic Carcinoma	HER 2+	Left DCIS (40)
Corso 2023 [9]	40	OC	BRCA1	c.5030_5033del p.(Thr1677Ilefs*2)	Left	MCHMD	TN	Right IDC (35)
Corso 2023 [9]	28	No family history	BRCA1	c.5030_5033del p.(Thr1677Ilefs*2)	Left	Mixed metaplastic Carcinoma	TN	
Corso 2023 [9]	27	BC	BRCA1	c.5179 A>T p.(Lys1727*)	Left	MCHMD	TN	
Corso 2023 [9]	32	BC/OC	BRCA1	c.5266dup p.(Gln1756Profs*74)	Right	Spindle cell carcinoma	TN	Left IDC (33)
Current case	52	BC	BRCA2	c.9676del p.(Tyr3226Ilefs*23)	Right	MCHMD	TN	

*: stop codon; BC: breast cancer; MBC: metaplastic breast cancer; IDC: infiltrating ductal carcinoma; OC: ovarian cancer; MCHMD: metaplastic carcinoma with heterologous mesenchymal differentiation; NOS: not otherwise specified; TN: triple negative; DCIS: ductal carcinoma in situ.

## Data Availability

The original contributions presented in this study are included in the article. Further inquiries can be directed to the corresponding authors.

## Abbreviations

The following abbreviations are used in this manuscript:

BC	Breast cancer
DCIS	Ductal carcinoma in situ
HPF	High power field
IDC	Infiltrating ductal carcinoma
MBC	Metaplastic breast carcinoma
MCHMD	Breast carcinoma with heterologous mesenchymal differentiation
NOS	Not otherwise specified
OC	Ovarian cancer
OGC	Osteoclast-like stromal giant cells
PARP	Poly (adenosine diphosphate [ADP]-ribose) polymerase
PV	pathogenic variant
TN	Triple-negayive
TNBCs	Triple-negative breast cancers
UCSs	Uterine carcinosarcomas
WHO	World Health Organization
